# Cumulative pulse methylprednisolone and its relation to disease activity, damage and mortality in systemic lupus erythematosus patients: A post hoc analysis of COMOSLE-EGYPT study

**DOI:** 10.1007/s10067-023-06858-4

**Published:** 2024-01-10

**Authors:** Nesreen Sobhy, Yasser Ezzat, Sherif M. Gamal, Shada A. Ghoniem, Sarah S. Nasr, Shaimaa Badran, Ahmed Soliman, Nermeen Ahmed Fouad

**Affiliations:** 1 Rheumatology Department, Cairo University, Cairo, Egypt; 2 Rheumatology Department, Fayoum University, Fayoum, Egypt; 3 Department of Cancer Epidemiology and Biostatistics, National Cancer Institute, Cairo University, Cairo, Egypt; 4 Department of Dermatology and Venereology, National Research Center Egypt, Cairo, Egypt

**Keywords:** Cumulative, Damage, Methylprednisolone, Mortality, Systemic lupus erythematosus

## Abstract

**Objective:**

To investigate the relation between cumulative intravenous methylprednisolone dose and disease activity, damage, and mortality among a group of Egyptian SLE patients.

**Patients and methods:**

This is a post hoc analysis of a retrospective multicenter COMOSLE study. Cumulative pulse methylprednisolone dose was abstracted from COMOSLE database. Patients with cumulative pulse dose of ≤ 3.0 g (median dose) were compared to those with cumulative dose of > 3.0 g regarding demographic data, Systemic Lupus Erythematosus Disease Activity Index (SLEDAI) and The Systemic Lupus International Collaborating Clinics/ACR Damage Index (SLICC) score as well as treatment received. Additionally, at 1.5, 3, 6, and 9 g of cumulative methylprednisolone, patients were compared regarding SLICC score and risk of mortality.

**Results:**

Patients who received > 3 g of methylprednisolone were statistically significantly younger at disease onset, had longer disease duration, higher SLEDAI score at last visit, and higher SLICC score (*p* = 003, *p* = 0.002, *p* = 0.004 and *p* =  < 0.001, respectively). Additionally, with every gram increase in the cumulative methylprednisolone, there was a significant increase in SLICC score by 0.169 (*B* = 0.169, CI = 0.122–0.216, *p*-value =  < 0.001) and an increased risk of mortality by 13.5% (hazard ratio (HR) = 1.135, CI = 1.091–1.180, *p*-value = 0.001). The best cutoff value of methylprednisolone dose at which damage may occur, ranged between 2.75 (with sensitivity of 81.4% and specificity of 33.9%) and 3.25 g (with sensitivity of 48.3% and specificity of 71.5%).

**Conclusion:**

With every gram increase in the cumulative methylprednisolone, there may be increase in damage and mortality, especially in doses exceeding the range of 2.75–3.25 g.

**Table:** 

**Key Points** *• Treatment of systemic lupus erythematosus should be with the least possible dose of steroids to decrease the risk of damage and mortality.* *• With every gram increase in the cumulative intravenous methylprednisolone there may be increase in damage and mortality.*

## Introduction

The management of SLE is often challenging aiming to control disease activity, enhance health-related quality of life, and minimize drug-related adverse events [1].

Organ damage in SLE tends to accumulate over time which occurs due to both the disease itself and its treatment. Both have a significant negative impact on life expectancy and quality of life of patients [2]. Assessment of accumulated damage due to SLE has been considered as an important outcome [3]

Corticosteroids are the mainstay of therapy for SLE and have been traditionally used to induce disease remission either as the sole agent or, more commonly, in combination with other immunosuppressants, with the route of administration and dosage schedule dependent on the severity and distribution of organ involvement, such therapy often achieves disease remission, but too many times at the cost of a large degree of damage [4, 5].

Intravenous methylprednisolone (IVMP) is one that continues to provoke considerable debate as to its precise role and as a rapid method of immunosuppression in SLE patients with organ and life-threatening manifestations [6], despite that the best dose, timing, and the situations in which this treatment should be given remain unclear [7].

The objective of this study is to investigate the relation between cumulative IVMP dose and disease activity, damage, and mortality among a group of Egyptian SLE patients and to predict the cumulative dose at which damage may start.

## Patients and methods

This study is a post hoc analysis of COMOSLE data. COMOSLE is a retrospective multicenter study in which medical records of 902 SLE patients were reviewed. Patients were attending rheumatology units in four Egyptian University hospitals and one private rheumatology center [8]. The study was conducted in accordance with the guidelines of the Declaration of Helsinki and was revised and ethically approved by the Research Ethics Committee of the Faculty of Medicine, Cairo University, with the approval number (N-157–2023).

Cumulative IVMP dose was abstracted from COMOSLE database for all patients; the median cumulative pulse of methylprednisolone received by the studied patients was 3 g; accordingly, patients were divided into 2 groups: those who received ≤ 3.0 g (group 1) and those who were given > 3.0 g (group 2). Comparison between the 2 groups was done regarding demographic data, SLEDAI [9], and SLICC scores [10] as well as the treatment received. In addition, at 1.5, 3, 6, and 9 g as cutoff doses of pulse methylprednisolone, patients were compared regarding damage, rate, and risk of mortality.

## Statistical analysis

Data management and analysis were performed using Statistical Package for Social Sciences (SPSS) vs. 26. Categorical data were described as numbers and percentages. Numeric data were checked for normality and were statistically described in terms of mean and standard deviation or median and range as appropriate. When comparing categorical data, chi-square test or Fisher’s exact test was used appropriately. For numeric data, independent Student’s *t*-test was performed for normally distributed variables, and the Mann–Whitney *U* test was used for non-normally distributed variables. The Kruskal Wallis test with pair-wise Bonferroni adjustment was used to test the SLICC damage index in relation to methylprednisolone cumulative dose. Multiple regression analysis was used with forward LR variable selection method to calculate the odds ratios (OR). A receiver operator characteristic (ROC) analysis was performed to predict the cumulative pulse methylprednisolone level at which damage may start to occur. Accuracy is measured by the area under the ROC curve (AUC). The area close to 1.0 represents a perfect test; an area close to 0.5 represents a worthless test. Survival analysis was done using Kaplan–Meier method for comparison between different pulse methylprednisolone, and log rank test was performed. Cox regression analysis was done using forward likelihood-ratio (LR) method for calculating hazard ratios (HR). Overall survival rates (OS) were calculated from the date of diagnosis to the date of death or last follow-up. All tests were two-tailed, *p*-values < 0.05 were considered statistically significant, and 95% confidence level was used.

## Results

This study enrolled 902 SLE patients, 832 were females and 70 were males. Their mean age was 32.5 ± 9.3 years, and their mean disease duration was 9.11 ± 6.26 years. Details of demographic data and clinical and laboratory features of the studied patients are comparable to those in COMOSLE-Egypt study [8].

Out of the studied patients, 676 patients received intravenous methylprednisolone. Those who received IVMP were further subdivided into 2 groups: group one (401 patients) who received ≤ 3 g and group two (275 patients) who received > 3 g, and were compared regarding demographic data, SLEDAI and SLICC score, some laboratory data as well as treatment received. This comparison showed that group (2) patients showed statistically significant higher SLEDAI at last visit and higher SLICC damage index (*p* = 0.004, p =  < 0.001, respectively). Details of such comparison are shown in Table 1.

**Table 1:** Demographic, SLEDAI, SLICC score, some laboratory data and treatment received of the studied groups

	(Group 1) (*n* = 401) *n* (%)	(Group 2) (*n* = 275) *n* (%)	*P*-value
Gender			
Female	379 (61.2)	240 (38.8)	0.001*
Male	22 (38.6)	35 (61.4)	
Age, mean (SD)	32.3 (9.0)	31.7 (8.7)	0.366
Age at Onset, mean (SD)	24.0 (8.9)	22.0 (8.2)	0.003*
Disease duration in years, median (range)	7 (0.5–26)	9 (0.5–30)	0.002*
SLEDAI			
At onset, median (range)	12.0 (0.0–47.0)	13.0 (1.0–49.0)	0.128
At last visit, median (range)	4.0 (0.0–34.0)	5.0 (0.0–27.0)	0.004*
SLICC score, median (range)	1 (0.0–7.0)	2 (0.0–10.0)	< 0.001*
Complement consumption at last visit (*n* = 770)	120 (50.2)	119 (49.8)	0.002*
ACL IgG (*n* = 613)	68 (48.2)	73 (51.8)	0.039*
ACL IgM (*n* = 526)	54 (47.4)	60 (52.6)	0.161
LAC (*n* = 536)	85 (61.6)	53 (38.4)	0.045*
Cumulative cyclophosphamide level, median (range)	5.9 (0.5–850.0)	6.0 (0.4–900.0)	0.528
Azathioprine	321 (57.4)	238 (42.6)	0.028*
Mycophenolate mofetil	96 (44.4)	120 (55.6)	< 0.001*
Steroid dose at last visit, median (range)	15.0 (2.0–60.0)	17.5 (2.5–60.0)	0.001*
Anti-malarials	377 (58.9)	263 (41.1)	0.356

*Statistically significant at < 0.05 level

*SD* standard deviation, *SLEDAI* SLE disease activity index, *SLICC* The Systemic Lupus International Collaborating Clinics/ACR Damage Index

Further analysis of SLICC damage subcategory that has a direct relation to glucocorticoids showed that for every gram increase in cumulative solumedrol, there is an observed increase in the risk of osteoporosis by 9.2% (OR = 1.092), avascular necrosis by 4.7% (OR = 1.047), cataract by 3.7% (OR = 1.037), and diabetes by 4.7% (OR = 1.047). Taken into consideration that the presence or absence of diabetes was not extracted from the patients’ medical record before SLE onset.

Analysis of SLICC damage according to cumulative IVMP dose (≤ 1.5, > 1.5 to 3, > 3 to 6, > 6 to 9, > 9 g) was performed. A significantly different median damage index was found across cumulative IVMP dose as shown in Table 2.

**Table 2:** SLICC damage index by methylprednisolone cumulative dose

Cumulative methylprednisolone dose^***^	*N*	SLICC median		Index (range)	*P*-value **
≤ 1.5	117	1	1	(0–7)	< 0.001*
> 1.5 to 3 ^ab^	284	0	1	(0–7)	
> 3 to 6 ^c^	177	1	1	(0–10)	
> 6 to 9 ^ab/d^	51	1	2	(0–8)	
> 9 g	47	3	3	(0–10)	

*Statistically significant at < 0.05 level; **Kruskal Wallis test;***On performing Bonferroni adjusted pair-wise comparisons; categories with different letters are significantly different from each other, while categories sharing the same letter showed NO statistical significance. This means that categories marked with “a” do not significantly differ from each other, but they differ from categories marked with “c” and “d,” e.g., ≤ 1.5 a does not differ from > 1.5 to 3 ab nor > 6 to 9 ab but differs from > 3 to 6 c and > 9 d. The same for > 1.5 to 3 ab, it differs from > 3 to 6 c and > 9 d but does not differ from ≤ 1.5 a nor > 6 to 9 ab/d, etc

Regression increases for the relation between SLICC index and IVMP cumulative dose showed that there was a significant positive relation with an increase in SLICC index by 0.169 for every gram increase in the cumulative IVMP (*B* = 0.169, CI = 0.122–0.216, *p*-value =  < 0.001).

Median follow-up time for all patients was 96.1 months (range = 4.0–12038.6), while median survival time was 456.5 months (CI = 202.0–711.1). Five years overall survival (OS) was 94.7% and 10 years OS was 89.3%.

Survival analysis in relation to IVMP cumulative dose (in grams) showed that there is an increased risk of mortality by 13.5% for every gram increase in the cumulative IVMP (hazard ratio (HR) = 1.135, CI = 1.091–1.180, *p*-value = 0.001).

**Fig. 1:**
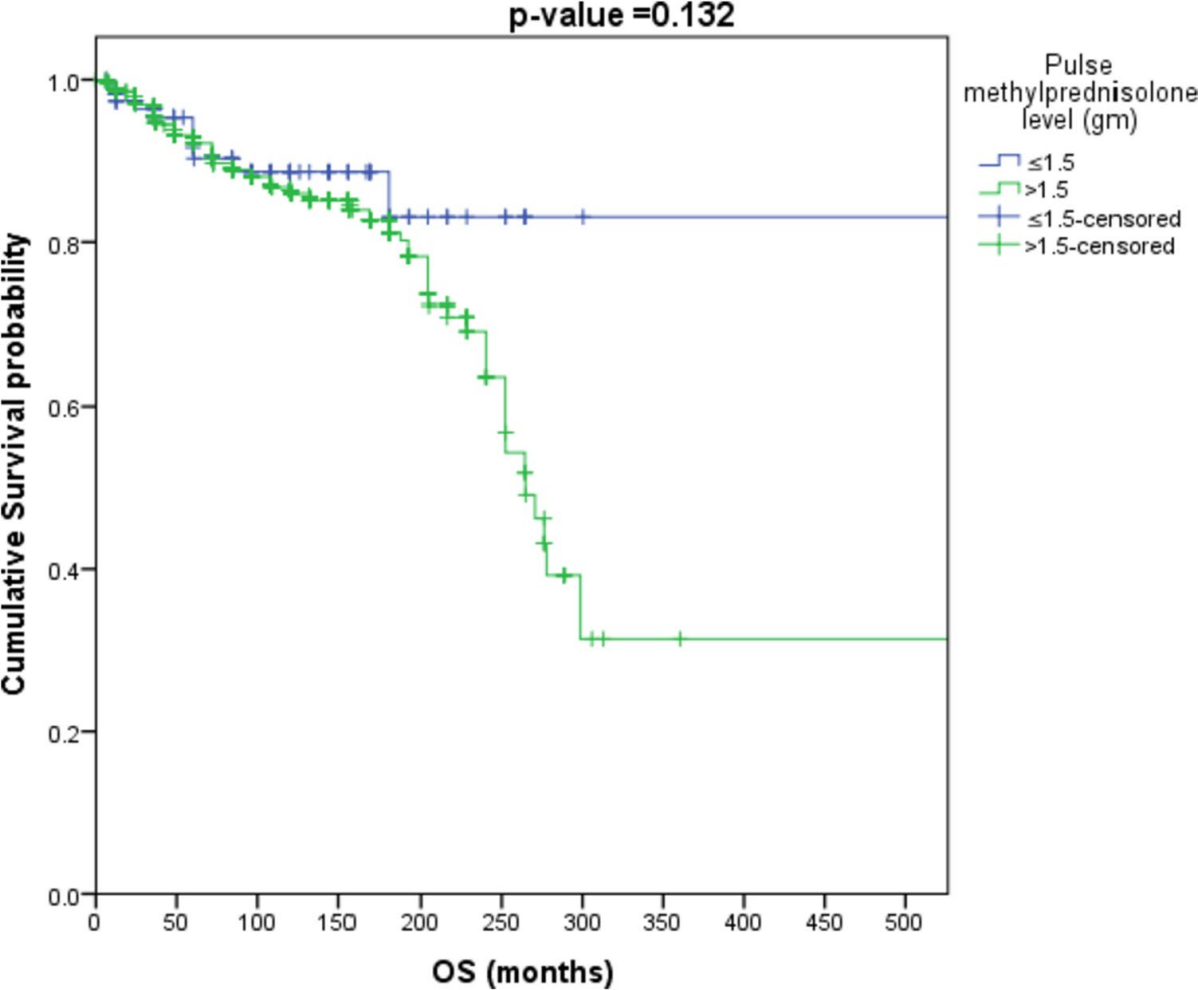
Survival analysis to those receiving cumulative methylprednisolone dose > 1.5 g and ≤ 1.5 g

**Fig. 2:**
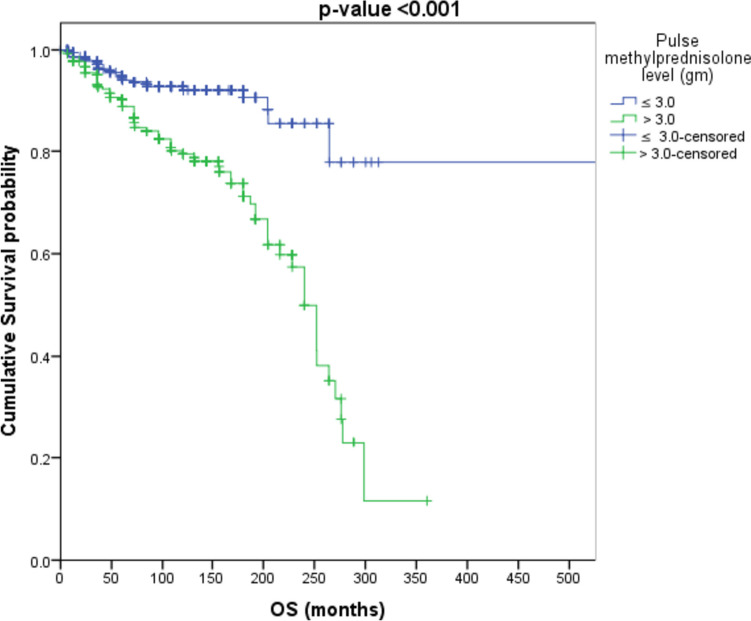
Survival analysis to those receiving cumulative methylprednisolone dose > 3 g and ≤ 3 g

IVMP cumulative dose was further classified into less or equal/more than 1.5, 3, 6, and 9 g. Survival analysis showed that patients with “cumulative IVMP > 1.5 g” have an increased risk of mortality by about 1.6 times as compared to patients with “cumulative IVMP ≤ 1.5 g” (HR = 1.611, *p*-value = 0.132), as shown in Fig. 1. Patients with “cumulative IVMP level > 3 g” have increased risk of mortality by about 3.4 times as compared to patients with “ cumulative IVMP ≤ 3 g”(HR = 3.419, *p*-value < 0.001) as shown in Fig. 2.

**Fig. 3:**
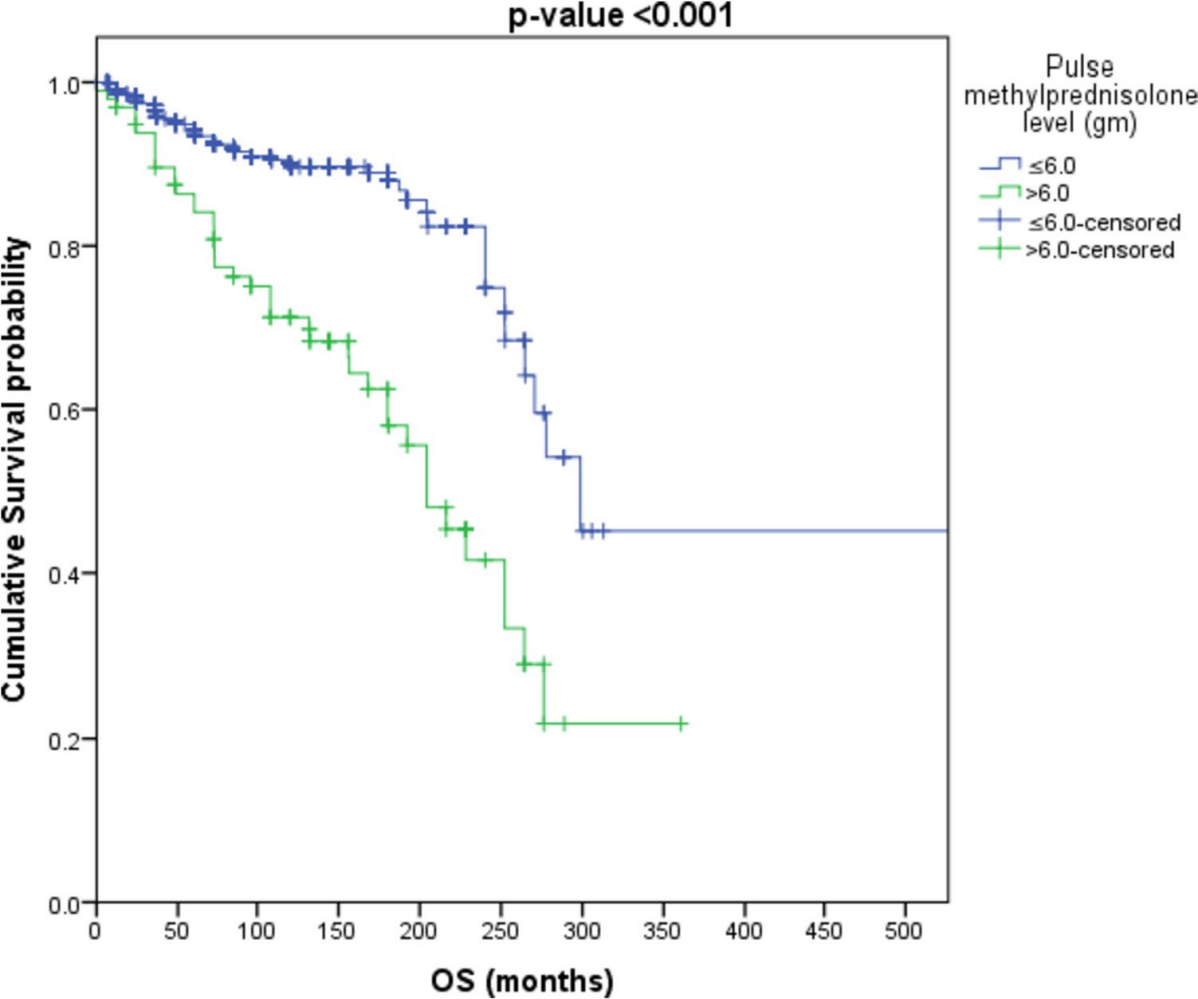
Survival analysis to those receiving cumulative methylprednisolone dose > 6 g and ≤ 6 g

**Fig. 4:**
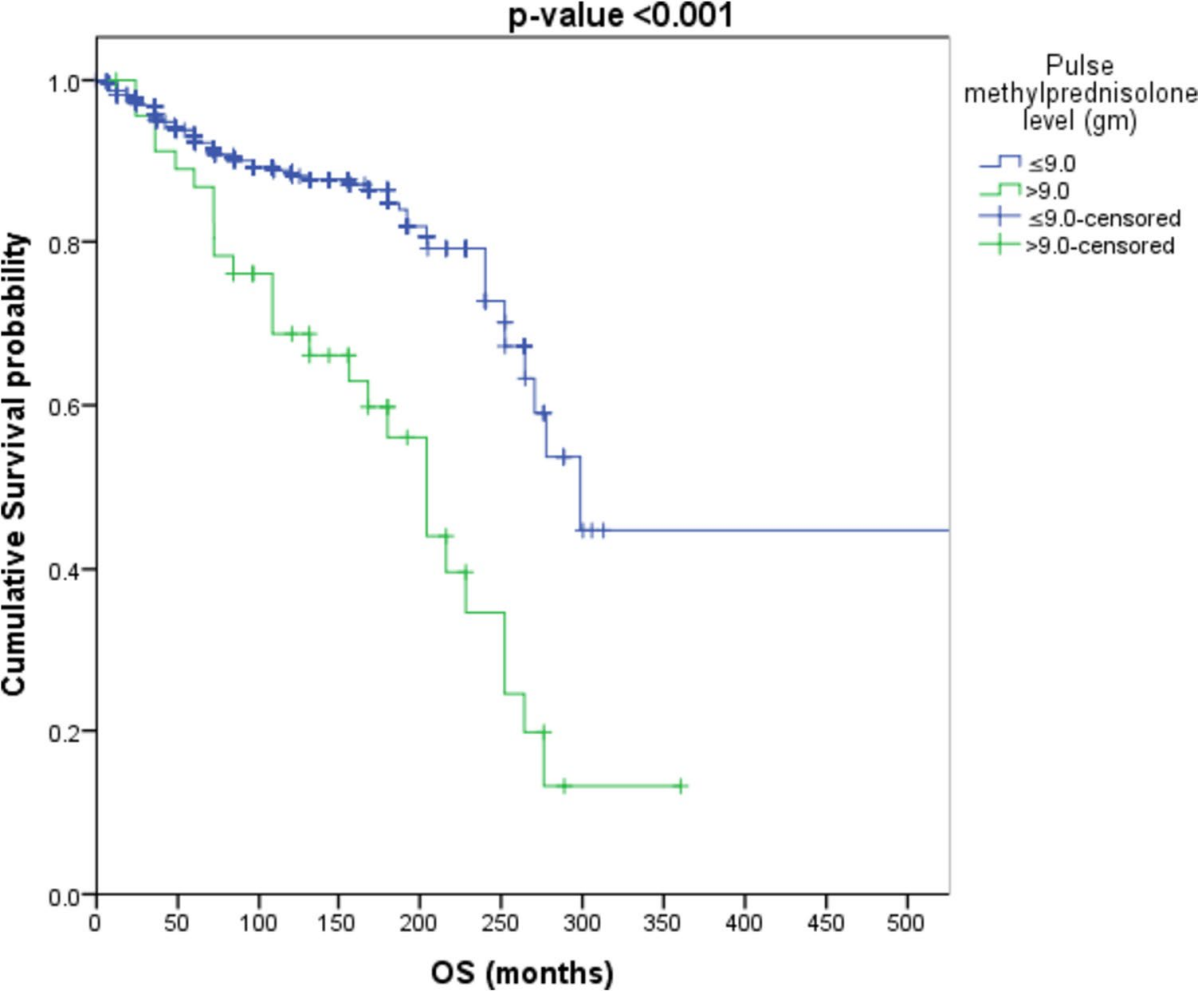
Survival analysis to those receiving cumulative methylprednisolone dose > 9 g and ≤ 9 g

Patients with “cumulative IVMP > 6 g” have an increased risk of mortality by about 3.2 times as compared to patients with” cumulative IVMP ≤ 6 g” (HR = 3.208, *p*-value < 0.001) as shown in Fig. 3. Patients with “cumulative IVMP level > 9 g” have an increased risk of mortality by about 3.1 times as compared to patients with “cumulative IVMP ≤ 9 g” (HR = 3.087, *p*-value < 0.001) as shown in Fig. 4.

**Fig. 5:**
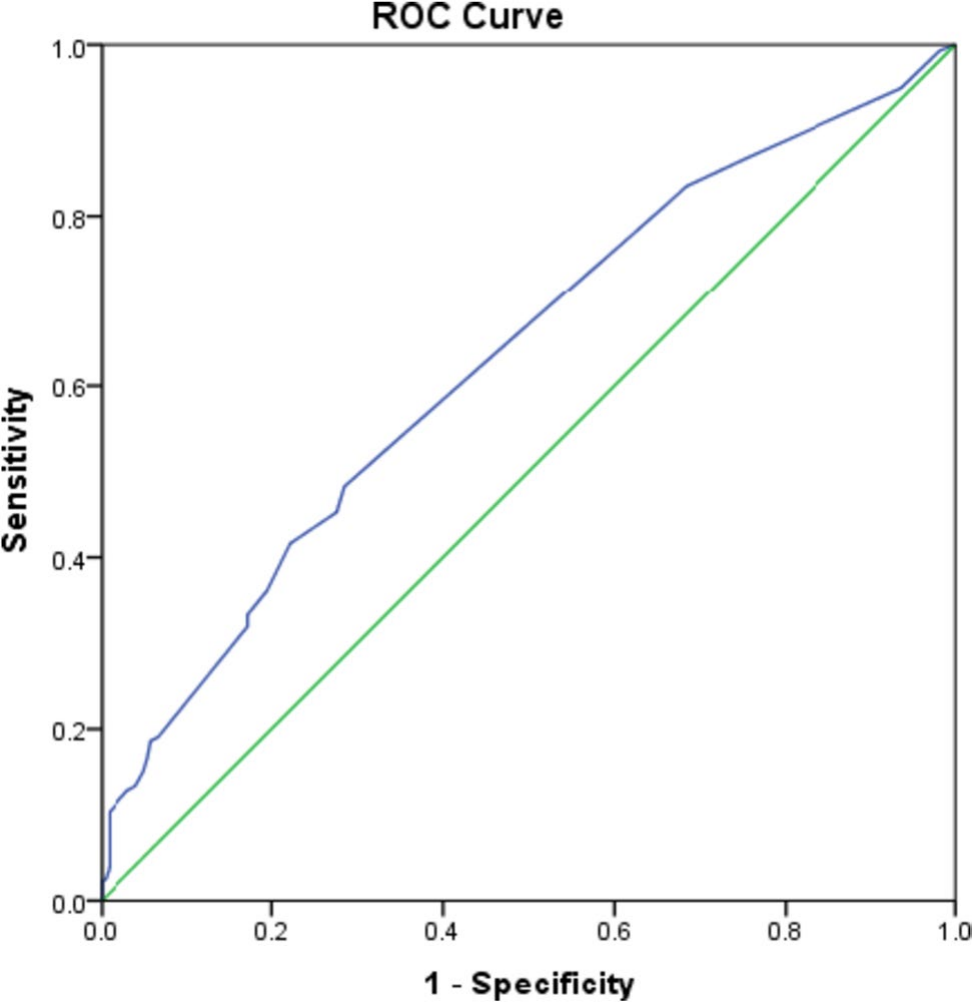
ROC curve for predicting SLICC damage according to cumulative pulse methylprednisolone dose. Best cut off range is between 2 values: 2.75 (with sensitivity of 81.4% and specificity of 33.9%), and 3.25 (with sensitivity of 48.3% and specificity of 71.5%)

ROC curve was done to predict the cumulative pulse methylprednisolone at which damage may start as shown in Fig. 5. We found that the best cutoff range is between 2 values: 2.75 (with sensitivity of 81.4% and specificity of 33.9%), and 3.25 (with sensitivity of 48.3% and specificity of 71.5%).

## Discussion

Glucocorticoids are a cornerstone in the management of SLE, often being inevitable especially in severe cases, aiming to halt disease progression. At the same time, they present a major cause of toxicity, thus remaining a major challenge, further attributing to and increasing the risk of possible damage in SLE [11]. Although many studies evaluated the impact of cumulative oral steroid on damage accrual and survival in lupus patients, few if any assessed the effect of cumulative pulse methylprednisolone. In general, low-dose pulse methylprednisolone often allows reduction of the oral steroid dose and is considered safer and less toxic than high-dose oral glucocorticoids [12]. However, repeated and higher cumulative pulse methylprednisolone doses may be associated with a higher risk compared to short, low-dose pulse administration. To our knowledge, this may be based on many factors; most importantly, patients requiring frequent and higher cumulative pulse have an active and poorly controlled disease course, usually receiving higher doses of oral steroids in addition to other immunosuppressives, which is reflecting the situation in our study group: patients treated with pulse doses > 3 g showed significantly higher oral steroid doses in the last visit, a significantly higher percentage of patients was receiving mycophenolate mofetil, also higher cumulative cyclophosphamide intake was detected, however not reaching a statistically significant level, in addition to a significantly higher SLEDAI at last visit. This is in accordance with Koelmeyer and his colleagues 2020, who described the association of more severe disease in SLE with higher disease activity across time, corticosteroid exposure, and damage accrual [13].

In this study, higher cumulative pulse methylprednisolone was associated with higher damage in lupus patients and that damage was expanded (increased) by 0.169 for every gram increase in the cumulative pulse level, which is in agreement with several studies that documented glucocorticoid-induced damage in lupus patients [14–18], describing up to 80% of organ damage in SLE being attributable to glucocorticoid use [19], with a clear dose-dependent relationship [4]. This also goes in line with the recommendation of limiting glucocorticoid use to the shortest possible time and the lowest dose possible [20]. The study by Badsha et al. (2003) confirmed that 1–1.5 g methylprednisolone versus 3–5 g methylprednisolone total dosage over 3 days may show equal efficacy, but with higher complications and risk of infection observed with the higher (3–5 g) dose [7].

A study by Ruiz-Arruza et al. (2014), evaluating the impact of pulse methylprednisolone on damage in SLE patients, showed contradictory outcomes to our results [12]. However, in their study, cumulative pulse was calculated over 4 years only, which may result in a lower cumulative dose compared to our study, in which we found that cumulative pulse methylprednisolone with doses lower than 2.75–3.25 g may not be associated with damage. Ugarte-Gil MF et al. (2021) reported that the differences in study populations and chronological periods, research designs and methodologies used, duration of disease and study observation as well as the different ways of expressing glucocorticoid exposure (daily, cumulative, oral vs parenteral) may lead to inconsistent conclusions [21].

Despite advancement in treatment and medical care and improved disease outcome, survival in lupus patients is still lower than the general population [22]. Many studies in the literature evaluated this topic and reported that the higher mortality rates in SLE patients are usually multifactorial, with higher doses and longer duration of corticosteroid therapy being one of the most important factors [23–26].

In the current study, survival analysis in relation to cumulative methylprednisolone level (in grams) showed that there is an increased risk of mortality by 13.5% for every gram increase in the cumulative Solumedrol level (hazard ratio (HR) = 1.135, CI = 1.091–1.180, *p*-value = 0.001). Such results are in consonance with the previously mentioned studies on corticosteroid and premature mortality in lupus patients, and in accord with the studies of Badsha et al. (2003) and Gamal et al. (2023), which showed that higher doses of pulse methylprednisolone may be associated with increased risk of adverse events and higher mortality [7, 27].

The prompt action of glucocorticoids especially methylprednisolone in dampening recurrent disease flare and in controlling active or difficult-to-treat lupus [28–31] may in our opinion encourage us to consider cumulative pulse methylprednisolone as rough predictor of frequent or recurrent disease flare and in turn higher risk of damage and mortality.

The major strength of our study is that it is one of the few analyses conducted in Arabic and African countries addressing the impact of cumulative pulse methylprednisolone on damage and mortality risk on lupus patients over a long follow-up period. One of the main limitations of the current study is the lack of cumulative oral steroid dose; however, in our opinion, accurate cumulative oral steroid calculation may be difficult in lupus patients with long follow-up periods, due to frequent fluctuations in doses with disease flares and remissions, lost follow-up of patients at some time intervals during the disease course, thus cumulative pulse methylprednisolone dose calculation which is usually administrated in the hospital might be much easier to calculate and can give a better index, if our results will be augmented by further studies. Also, blood sugar level before systemic lupus erythematosus onset was not recorded.

## Conclusion

Control of disease activity should be achieved with the least possible dose of intravenous methylprednisolone aiming not to exceed 3.25 g as a cumulative dose throughout the disease to decrease the risk of damage and mortality being cognizant of the cutoff value calculated in our study.
